# Copper and cadmium in bottom sediments dredged from Wyścigi Pond, Warsaw, Poland—contamination and bioaccumulation study

**DOI:** 10.1007/s10661-015-4945-0

**Published:** 2015-11-10

**Authors:** Małgorzata Wojtkowska, Ewa Karwowska, Iwona Chmielewska, Kundyz Bekenova, Ewa Wanot

**Affiliations:** Faculty of Environmental Engineering, Warsaw University of Technology, Nowowiejska 20, 00-653 Warsaw, Poland

**Keywords:** Heavy metals, Surface water, Bottom sediment, Vegetation

## Abstract

This research covered an evaluation of the copper and cadmium concentrations in bottom sediments dredged from one of the ponds in Warsaw. The samples of sediments, soil, and plants were analyzed in terms of Cu and Cd content. The research concerned the heap of dredged bottom sediments from Wyścigi Pond, Warsaw, Poland. Two boreholes were made to obtain sediment cores with depths of A 162.5 cm and B 190.0 cm. The cores were divided into 10 sub-samples with a thickness of about 15–20 cm. A control sample of soil was taken from the horse racecourse several hundred meters away from the heap. The vegetation was sampled directly from the heap. The predominating plants were tested: *Urtica dioica*, *Glechoma hederacea*, *Euonymus verrucosus*, and *Drepanocladus aduncus*. A control sample of *U. dioica* taken outside of the heap was also tested. The commercial PHYTOTOXKIT microbiotest was applied to evaluate the influence of heavy metal-contaminated sediments (used as soil) on germination and growth of the chosen test plants. The analyses of cadmium and copper concentrations revealed that the metal concentration in sediments was diverse at different depths of sampling, probably reflecting their concentration in stored layers of sediments. Moreover, the metal content in core A was four to five times lower than that in core B, which reveals heterogeneity of the sediments in the tested heap. In core A, the copper concentration ranged from 4.7 to 13.4 mg/kg d.w. (average 8.06 ± 0.71 mg/kg d.w.), while in core B, it ranged from 9.2 to 82.1 mg/kg d.w. (average 38.56 ± 2.6 mg/kg d.w.). One of the results of the heavy metal presence in soils is their bioaccumulation in plants. Comparing plant growth, more intensive growth of roots was observed in the case of plants growing on the control (reference) soil than those growing on sediments. The intensive development of both primary and lateral roots was noticed. During this early growth, metal accumulation in plants occurred.

## Introduction

Surface water monitoring in Warsaw, Poland, confirmed a severe anthropogenic contamination (Wojtkowska 2013). One of the problems are significant amounts of heavy metals which are detected in bottom sediments dredged from water reservoirs during their restoration (Yao and Gao 2007; Wojtkowska 2011).

Wyścigi Pond is a flow reservoir with a retention capacity of 17,671 m^3^. In 2006, the pond was reconstructed in order to restore its capability for water retention and landscape value. As a result, a large amount of bottom sediments was excavated and stored in 6-m-high heaps at Horse Racing Służewiec area.

It was revealed that the sediments consist of some anthropogenic, organic, fluvial, and glaciofluvial materials. During the recultivation, they were mixed and the sequence of storage of different portions of the material is unknown. Therefore, the chemical composition as well as the distribution of contaminants within the heaps changes depending on individual properties of the bottom sediment at the place (technical documentation, not published).

After some years of storage, the heap material is still waste, although, due to its physical and chemical properties, it could be potentially used as a soil-like component for land reclamation and agricultural purposes. However, it is suspected that the sediments of the Wyścigi Pond may contain increased concentrations of some metals, especially copper and cadmium. Depending on environmental conditions, the metals can be released from the heap and pollute the soil and ground waters (Jumbe and Nandini 2009).

Copper and cadmium are characteristic of anthropogenic contamination. In the environment, copper occurs usually in the form of Cu^2+^ cations although it is also able to create complex anions and cations, depending on environmental conditions (Table 1). In soils contaminated by copper mining and smelting, the concentration of Cu reaches hundreds of milligrams per kilogram of soil (Krajewski 1993). Cadmium tends to accumulate in surface layers of soil due to its strong sorption by clay minerals and organic substances (Gorlach 1995), although it is more mobile and bioavailable compared with other divalent ions in soil (Alloway and Ayres 1999). The sorption of cadmium in soil increases with increased soil pH (more intensively than in the case of Zn, Cu, or Pb) (Smal et al. 1998; Kabata-Pendias 2000; Kalembasa and Pakuła 2006). Zinc and lead smelters are the most significant industrial sources of cadmium (Kabata-Pendias 2000).

**Table 1:** Main forms of copper and cadmium in soils (Malinowska 2008)

Metal	Acidic soils	Anaerobic soils	Alkaline soils	Aerated soils
Cd(II)	Cd^2+^, CdSO_4_^0^, CdCl^+^	Cd, CdS	Cd^2+^, CdCl^+^, CdSO_4_^0^, CdHCO_3_^+^	Cd(OH)_2_, CdCO_3_
Cu(II)	Org., Cu^2+^, CuCl^+^	Cu, CuS, Cu_2_S	CuCO_3_0, org., CuHCO_3_	CuO, CuCO_3_, Cu_2_(OH)_2_CO_3_

## Methods

This research covered an evaluation of copper and cadmium concentrations in bottom sediments dredged from one of the ponds in Warsaw, the study on Cu and Cd bioaccumulation in heap vegetation, as well as the influence of sediments on germination and early growth of chosen plants.

## Samples

**Fig. 1:**
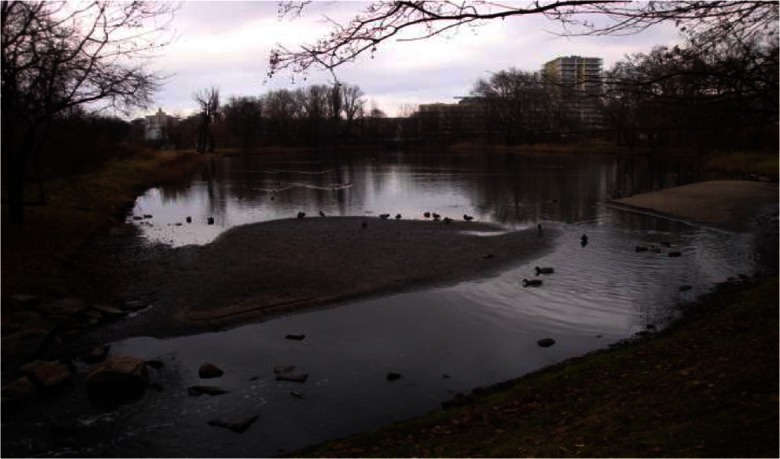
Wyścigi Pond, Warsaw, Poland—a source of bottom sediments

**Fig. 2:**
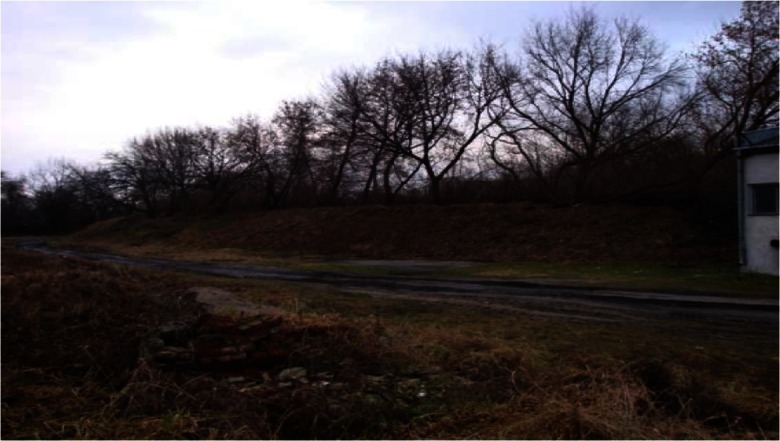
A tested heap of dredged bottom sediments

The research concerned the heap of dredged bottom sediments from Wyścigi Pond, Warsaw, Poland (Figs. 1 and 2).

**Fig. 3:**
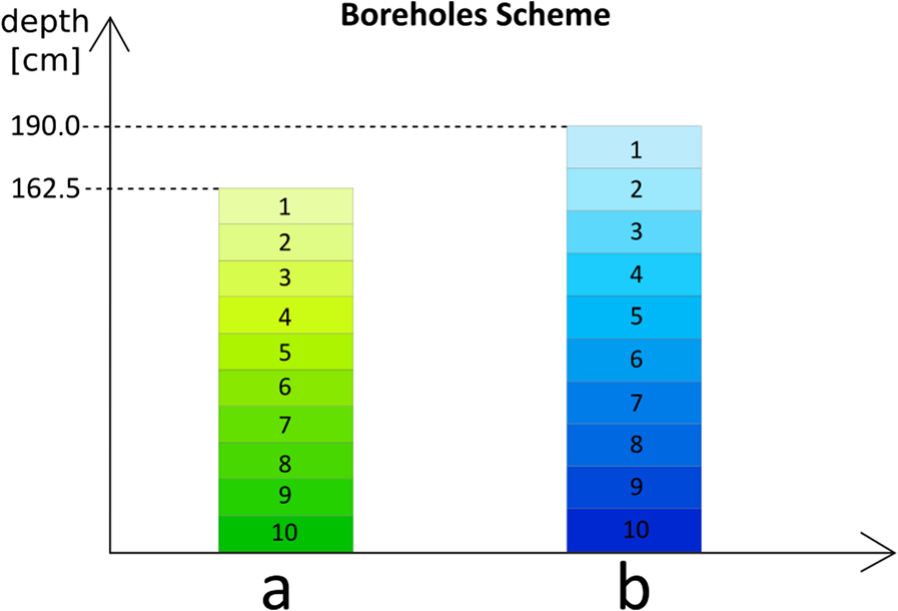
Boreholes scheme

The samples of bottom sediments were collected using a drilling metal probe. Two boreholes were made to obtain sediment cores at depths of A 162.5 cm and B 190 cm. The cores were divided into 10 sub-samples with a thickness of about 15–20 cm (Fig. 3). The control sample was soil from the horse racecourse, several hundred meters from the heap, with a thickness of about 15–20 cm.

The vegetation was sampled directly from the heap. The predominating plants were tested: *Urtica dioica*, *Glechoma hederacea*, *Euonymus verrucosus*, and *Drepanocladus aduncus*. A control sample of *U. dioica* from outside the heap was also tested.

The samples of sediments, soil, and plant biomass were analyzed in terms of Cu and Cd content. The samples of sediments, soil, and plants were dried at room temperature to constant weight. The metal concentration was determined using atomic absorption spectrometry (AAS). Total metal concentrations in all samples (sediment, soil, and plants) were determined by means of total extraction with HNO_3_ and HClO_4_ (1:3) in a Teflon bomb. All cations were analyzed using flame atomic absorption spectroscopy (FAAS) type Philips PU9100. Trace quantities of metals were determined by means of graphite furnace atomic absorption spectroscopy. Metal concentrations in the supernatant liquid were measured with flame atomic absorption spectrometry (FAAS). The same procedure without samples was used as a control. Three measurements were conducted for each sample. Quality assurance and quality control (QA/QC) for metals in sediment samples were estimated by determining metal concentrations in the Merck Standard solutions (Merck, Darmstadt, Germany). Blank determinations were carried out for each analysis batch. Reference sediment samples (BCR 701 Lake Sediment) were analyzed to determine the accuracy of the analysis. The detection limit was calculated based on the estimated instrumental detection limit, assuming that 1 g of a sample is digested or diluted to 100 mL. Detection limits (mg/kg of dry matter) for Cu and Cd were 0.003 and 0.001, respectively. The precision of the analytical procedures was calculated as relative standard deviation (RSD) and ranged from 5 to 10 %. It was calculated based on the standard deviation divided by the mean. Investigated metals’ rates of recovery were 90 ± 10 %. Chemicals, solutions, and reagents were of analytical grade. All glassware before use was washed with distilled water, rinsed in nitric acid (30 %), and then washed with deionized water and air-dried.

The commercial PHYTOTOXKIT microbiotest was applied to evaluate the influence of heavy metal-contaminated sediments (used as a soil) on germination and growth of chosen test plants: *Sorghum saccharatum*, *Sinapsis alba*, and *Lepidium sativum*. After 3 days of growth in the dark in 25 °C, the growth parameters as well as cadmium and copper content in plants were determined.

The plants from the PHYTOTOXKIT test were also analyzed in terms of Cu and Cd content. For *Sinapsis alba*, the metal concentration in 3-day-old plants was compared with that of the plants cultivated for 4 weeks in laboratory conditions on the sediments taken from the heap.

## Results

**Fig. 4:**
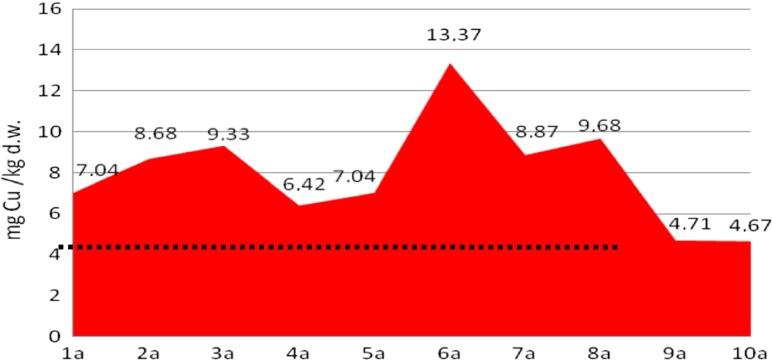
Copper content in the bottom sediment heap—core profile A, compared with background level (*dots*)

**Fig. 5:**
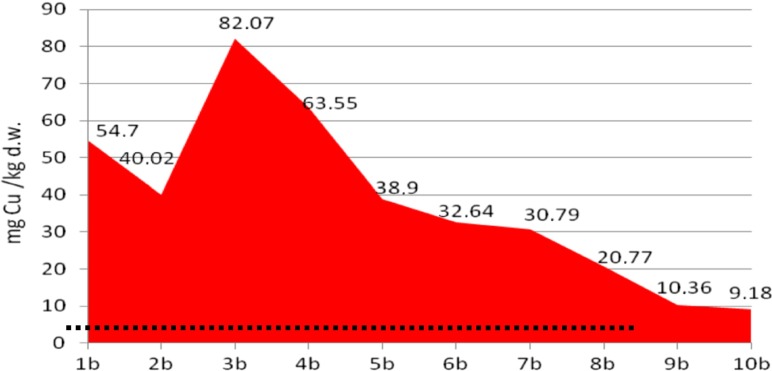
Copper content in the bottom sediment heap—core profile B, compared with background level (*dots*)

**Fig. 6:**
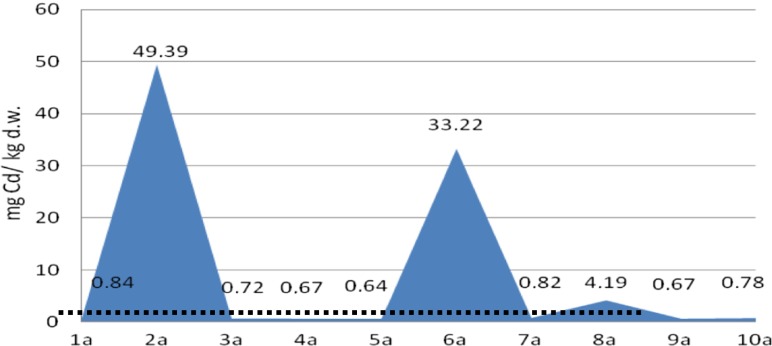
Cadmium content in the bottom sediment heap—core profile A, compared with background level (*dots*)

**Fig. 7:**
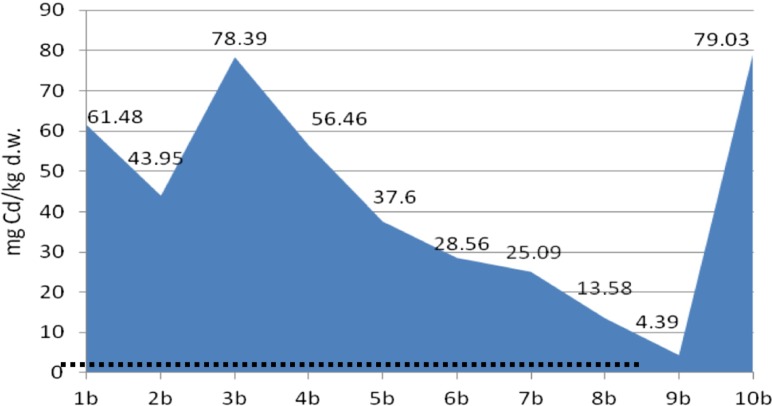
Cadmium content in the bottom sediment heap—core profile B, compared with background level (*dots*)

The analyses of cadmium and copper concentrations revealed that the metal concentration in sediments was diverse at different depths of sampling, probably reflecting their concentration in stored layers of sediments. Moreover, the metal content in core A was four to five times lower than that in core B, which reveals heterogeneity of the sediments in the tested heap (Figs. 4, 5, 6, and 7).

It was determined that in the core B profile, the concentration of metals decreased with depth of sampling (despite 10b sub-sample for cadmium). The effect was not observed in the core A profile, which was less metal loaded.

In core A, copper concentration ranged from 4.7 to 13.4 mg/kg d.w. (average 8.06 ± 0.71 mg/kg d.w.), while in core B, it ranged from 9.2 to 82.1 mg/kg d.w. (average 38.56 ± 1.54 mg/kg d.w.), while in the background concentration in soil (control sample), the Cu content did not exceed 4.49 mg/kg d.w.

The concentration of cadmium in core A was very low in most of the sub-samples, despite 2a and 6a, in which it was 49.39 and 33.22 mg/kg d.w, respectively. In core B, the cadmium content was much more higher, reaching 79.03 mg/kg d.w. (average 43.66 ± 2.6 mg/kg d.w.), while in the background concentration in soil (control sample), the Cd content did not exceed 1 mg/kg d.w.

According to the Polish Minister of Environment Regulation, the limit values for the Cu and Cd content in dredged sediments are 150 and 7.5 mg/kg of dry weight, respectively. In bottom sediments in the tested heap, the limit value for Cu content was not exceeded, but the concentration of cadmium was up to 10 times higher compared with the limit value.

The cadmium concentration, especially in core B, was more than 20 times higher in comparison with the background value; therefore, according to the classification of bottom sediments based on the geochemical criterion, the sediments from the heap belong to class III (contaminated) (Bojakowska and Sokołowska 1998).

Also, according to US EPA classification, the sediments should be considered as strongly polluted (Migaszewski and Gałuszka 2007).

The ecotoxicological criteria of sediment classification based on threshold effect level (TEL) and probable effects level (PEL) values were applied. The PEL value for cadmium (3.5 mg/kg) was exceeded in the case of core B and in three sub-samples from core A. The TEL value for copper (36 mg/kg) was exceeded in five samples. Based on this, the probable toxic effect of cadmium and copper in concentrations present in tested sediments may be observed (MacDonald et al. 2000).

The stored sediments are of potential use as a kind of soil. However, the heavy metal concentration should be considered. The typical concentrations of Cu and Cd in the surface layer of soils in Poland are presented in Table 2. The critical metal concentrations for different soil contamination levels are shown in Table 3.

**Table 2:** Heavy metal concentrations in the surface layer (0–20 cm) of soils in Poland [mg/kg s.m.] (Buczkowski et al. 2002)

Metal	Sandy soils		Clay and dusty soils		Organic soils	
	Range	Average	Range	Average	Range	Average
Cd	0.08–1.6	0.3	0.15–1.6	0.4	0.01–0.1	0.05
Cu	1–25	6.0	5–60	15.0	1–110	5.00

**Table 3:** Critical metal concentrations in the surface layer of the soil (0–20 cm) for different contamination levels—according to the Institute of Soil Science and Plant Cultivation (Kabata-Pendias et al. 1993)

Metal	Type of soil	Soil contamination [mg/kg]					
		Level 0	Level I	Level II	Level III	Level IV	Level V
Cd	A	0.3±	1.0	2.0	3.0	5.0	>5.0
	B	0.5±	1.5	3.0	5.0	10.0	>10.0
	C	1.0±	3.0	5.0	10.0	20.0	>20.0
Cu	A	15±	30	50	80	300	>300
	B	25±	50	80	100	500	>500
	C	40	70	100	150	750	>750

The contamination levels are characterized as follows (Buczkowski et al. 2002): *Level 0* soils without contamination (natural metal content), *Level I* soils with increased content of trace metals (limited application for cultivation of vegetables designed for children), *Level II* weakly contaminated soils (some plants should not be cultivated), *Level III* medium-contaminated soils (threat of plant contamination, heavy metal content in biomass should be monitored, mainly industrial plants and grass are recommended), *Level IV* strongly contaminated soils (should not be used in agriculture, some recultivation techniques may be applied), *Level V* extremely contaminated soils (should not be used in agriculture, recultivation recommended)

*A* very light soils, and light soils of low pH, *B* light soils with neutral pH and medium soils with low pH and organic matter content below 10 %, *C* medium and heavy soils, slightly acidic or neutral, soils of organic matter content more than 10 %

Compared with the presented values, the sediments dredged from Wyścigi Pond can be classified as soil belonging to level V, because of extremely high concentration of cadmium.

**Fig. 8:**
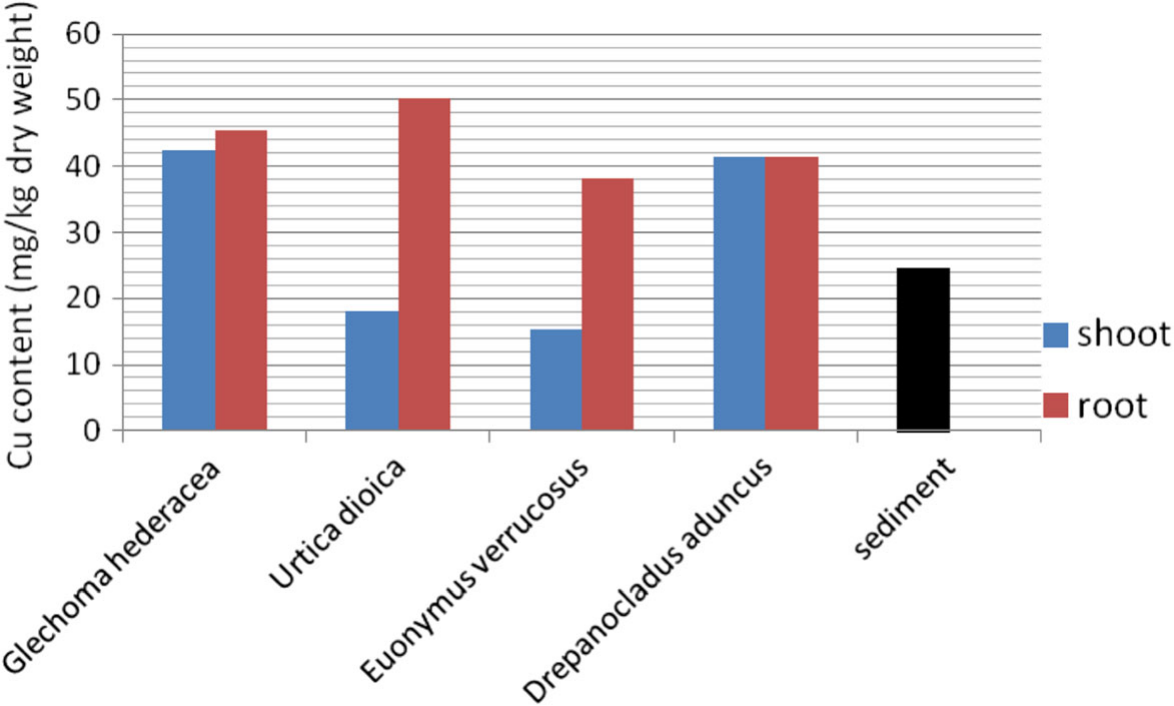
Copper concentration in heap vegetation compared with that of the sediment

**Fig. 9:**
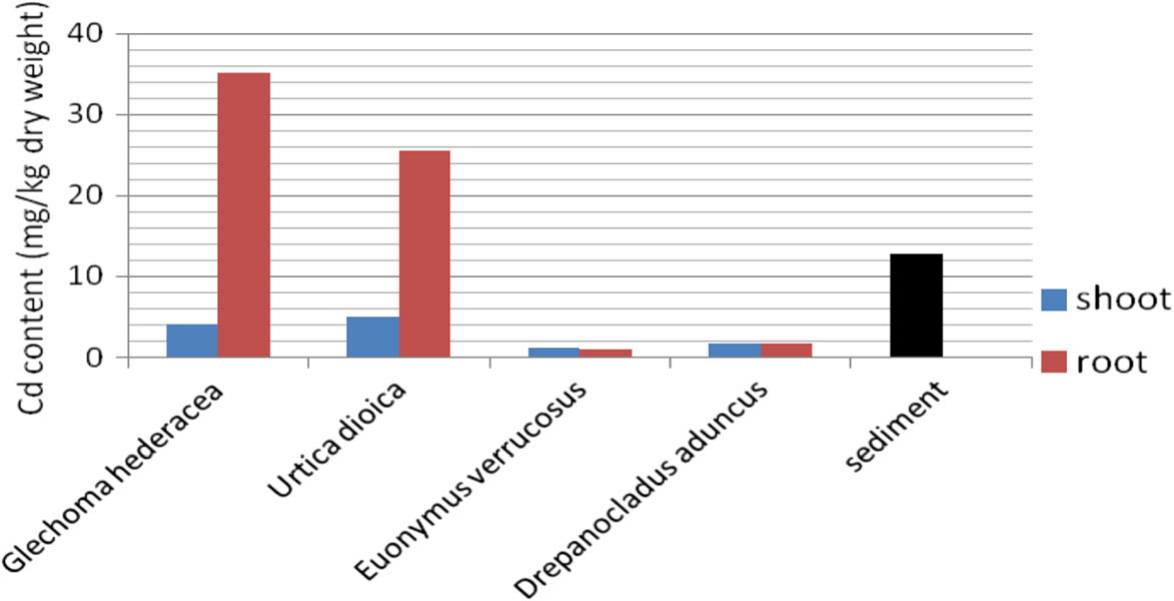
Cadmium concentration in heap vegetation compared with that of the sediment

One of the effects of the presence of heavy metals in soils is their bioaccumulation in plants. The results of the cadmium and copper content in vegetation samples taken from the heap are presented in Figs. 8 and 9, compared with the metal content in sediment at the place of their growth.

The concentration of Cu in roots of plants predominating on the surface of the heap was significantly higher than in the sediment. For two plants: *G. hederacea* and *U. dioica*, copper was accumulated also in aboveground parts. For these two species, the intensive cadmium accumulation in roots was also observed, with the content exceeding Cd concentration in sediments.

The preferential accumulation of tested metals in plant roots was described by Lityński and Jurkowska (1982) and Buczkowski et al. (2002).

**Fig. 10:**
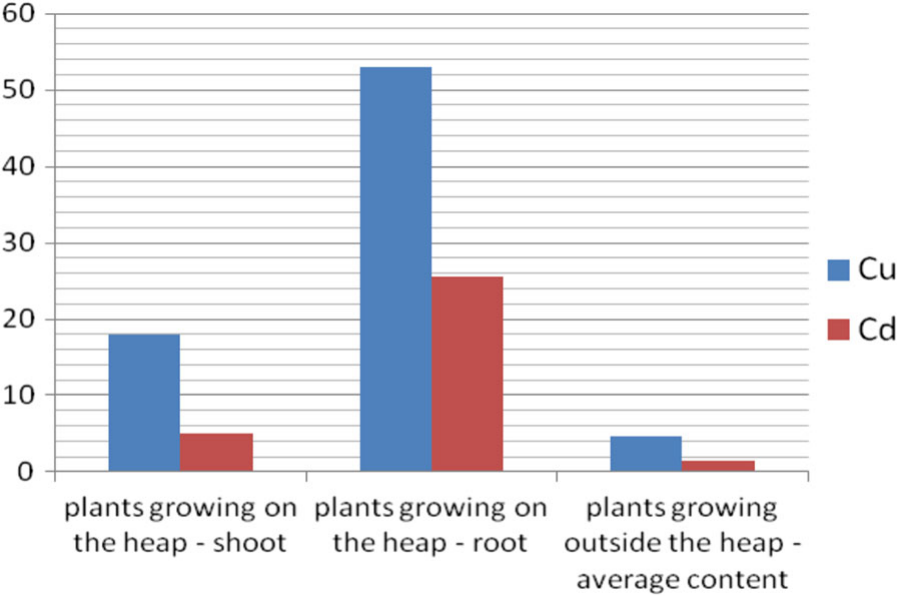
Average concentrations of Cu and Cd in *Urtica dioica* growing on the heap and at outside the heap

The observed effect was confirmed by the results of the analysis of the metal content in plants growing on the sediments compared with plants from outside the heap (Fig. 10).

In Table 4, the critical values of metal content in plants designated for different purposes are presented, according to the Institute of Soil Science and Plant Cultivation. According to them, the plants from the heap are strongly contaminated, especially in the case of cadmium (the lower limit of Cd for fodder plants was exceeded up to 60–70 times).

**Table 4:** Critical values of Cd and Cu in plants depending on their application (Kabata-Pendias et al. 1993)

Metal	Metal content in plants [mg/kg d.w.]		
	Food purposes	Industrial	Fodder
Cd	<0.15	<0.5	>0.5
Cu	<20	25–30	>30

**Fig. 11:**
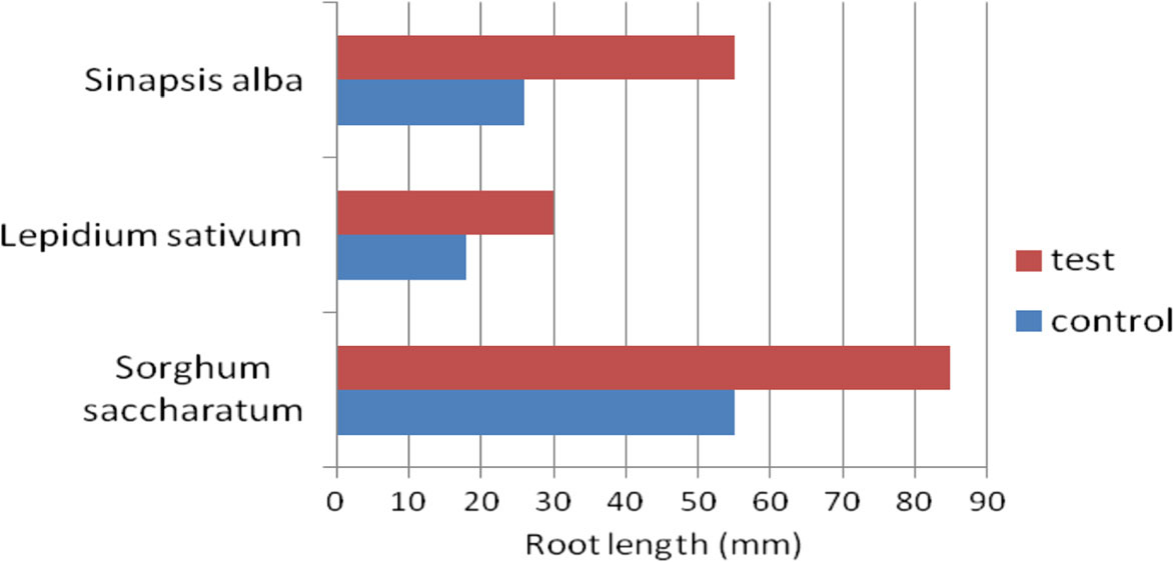
The influence of the sediments on root elongation in PHYTOTOXKIT test

Cadmium and copper are metals of potential toxic impact on the environment. It was suggested that metal-loaded sediments used as soil may influence the germination and growth of plants. To verify the thesis, the PHYTOTOXKIT microbiotest was carried out, using the heap sediments as soil for plant growth compared with standardized test soil (Table 5, Fig. 11).

**Table 5:** An inhibition of seed germination during the plant growth on sediments, compared with the reference soil

Test plant	The inhibition of seed germination (%)
*Sorghum saccharatum*	22.2
*Lepidium sativum*	22.2
*Sinapsis alba*	10.0

**Fig. 12:**
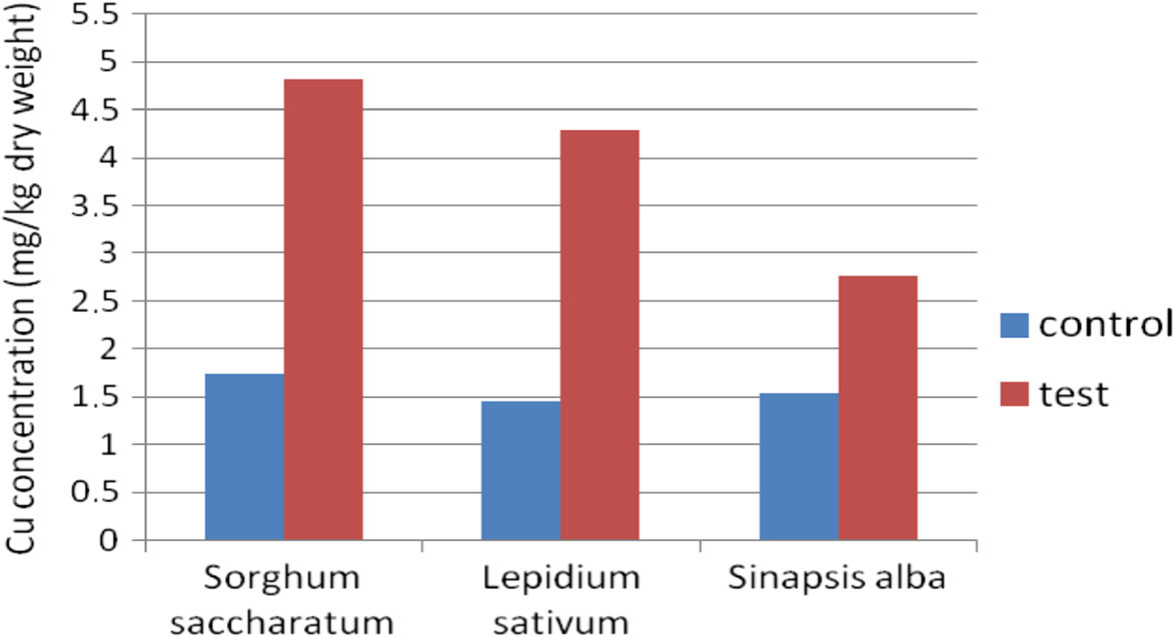
Copper accumulation in plants after PHYTOTOXKIT test

**Fig. 13:**
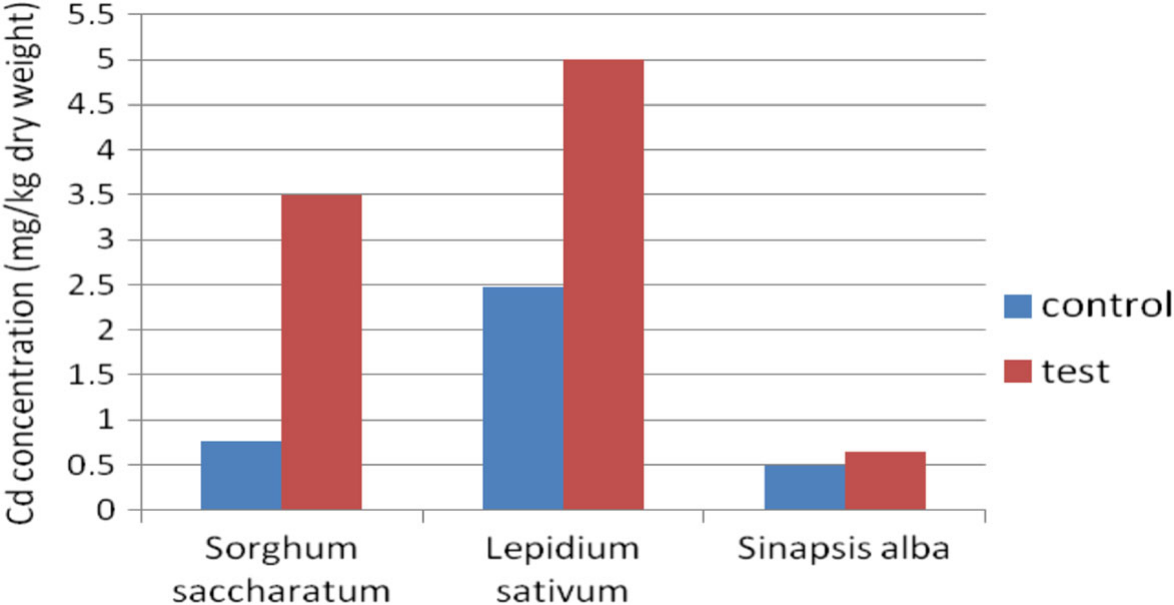
Cadmium accumulation in plants after PHYTOTOXKIT test

In the case of plants growing on sediments, the most intensive growth of roots was observed compared with plants growing on control (reference) soil. The intensive development of both primary and lateral roots was noticed. During this early growth, the metal accumulation in plants occurred (Figs. 12 and 13)

**Fig. 14:**
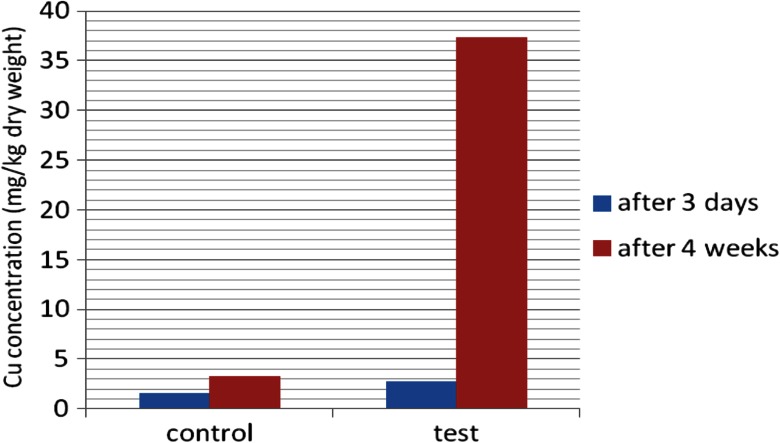
Cu concentration in *Sinapsis alba* after 3 days and 4 weeks of growth on metal-loaded bottom sediments

**Fig. 15:**
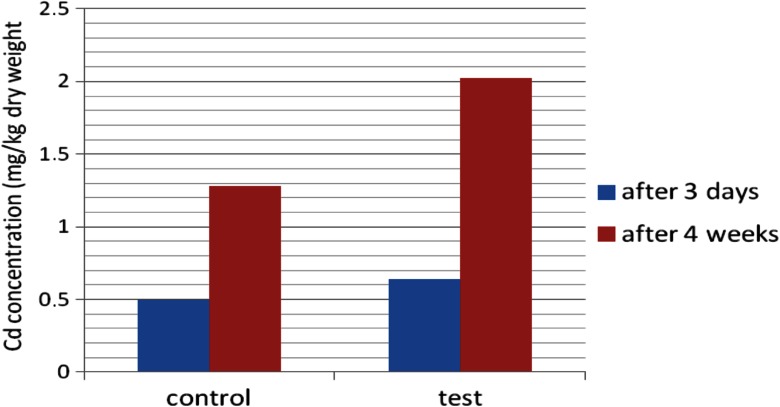
Cd concentration in *Sinapsis alba* after 3 days and 4 weeks of growth on metal-loaded bottom sediments

After the PHYTOTOXKIT test, the lowest concentrations of metals were noticed for *Sinapsis alba*. Because of this, the plant was then cultivated on sediments from the heap and reference soil, for 4 weeks in laboratory conditions. Then, the concentrations of Cu and Cd were determined and compared with those obtained after 3 days (Figs. 14 and 15). The significant increase in metal content in *Sinapsis alba* plants was observed after prolonged growth on contaminated ground, especially in the case of copper.

## Conclusions

The research revealed that the heap of bottom sediments dredged from Wyścigi Pond in Warsaw is strongly contaminated with cadmium and less significantly with copper. The pollution level confirms the probable long-term anthropogenic impact resulting in sediment degradation and making them useless for agricultural purposes. It should be stressed that the storage of cadmium-loaded sediments may be a cause of the secondary contamination of nearby soils and groundwaters, due to the mobility of the metal in soil environment. The vegetation on the heap contains comparatively high concentrations of both cadmium and copper due to the bioaccumulation process. The metals present in sediments are able to influence the germination and growth of the plants. Therefore, the immediate recultivation of the heap is recommended.
